# Reduced visual surround suppression in schizophrenia shown by measuring contrast detection thresholds

**DOI:** 10.3389/fpsyg.2014.01431

**Published:** 2014-12-10

**Authors:** Ignacio Serrano-Pedraza, Verónica Romero-Ferreiro, Jenny C. A. Read, Teresa Diéguez-Risco, Alexandra Bagney, Montserrat Caballero-González, Javier Rodríguez-Torresano, Roberto Rodriguez-Jimenez

**Affiliations:** ^1^Departmento de Psicología Básica I (Procesos Básicos), Complutense University of MadridMadrid, Spain; ^2^Institute of Neuroscience, Newcastle UniversityNewcastle upon Tyne, UK; ^3^Department of Psychiatry, Instituto de Investigación Hospital 12 de Octubre (i+12)Madrid, Spain; ^4^Centro de Investigación Biomédica en Red de Salud Mental (CIBERSAM)Madrid, Spain

**Keywords:** visual surround suppression, schizophrenia, GABA, psychophysics, inhibitory connections

## Abstract

Visual perception in schizophrenia is attracting a broad interest given the deep knowledge that we have about the visual system in healthy populations. One example is the class of effects known collectively as visual surround suppression. For example, the visibility of a grating located in the visual periphery is impaired by the presence of a surrounding grating of the same spatial frequency and orientation. Previous studies have suggested abnormal visual surround suppression in patients with schizophrenia. Given that schizophrenia patients have cortical alterations including hypofunction of NMDA receptors and reduced concentration of GABA neurotransmitter, which affect lateral inhibitory connections, then they should be relatively better than controls at detecting visual stimuli that are usually suppressed. We tested this hypothesis by measuring contrast detection thresholds using a new stimulus configuration. We tested two groups: 21 schizophrenia patients and 24 healthy subjects. Thresholds were obtained using Bayesian staircases in a four-alternative forced-choice detection task where the target was a grating within a 3∘ Butterworth window that appeared in one of four possible positions at 5∘ eccentricity. We compared three conditions, (a) target with no-surround, (b) target embedded within a surrounding grating of 20∘ diameter and 25% contrast with same spatial frequency and orthogonal orientation, and (c) target embedded within a surrounding grating with parallel (same) orientation. Previous results with healthy populations have shown that contrast thresholds are lower for orthogonal and no-surround (NS) conditions than for parallel surround (PS). The log-ratios between parallel and NS thresholds are used as an index quantifying visual surround suppression. Patients performed poorly compared to controls in the NS and orthogonal-surround conditions. However, they performed as well as controls when the surround was parallel, resulting in significantly lower suppression indices in patients. To examine whether the difference in suppression was driven by the lower NS thresholds for controls, we examined a matched subgroup of controls and patients, selected to have similar thresholds in the NS condition. Patients performed significantly better in the PS condition than controls. This analysis therefore indicates that a PS raised contrast thresholds less in patients than in controls. Our results support the hypothesis that inhibitory connections in early visual cortex are impaired in schizophrenia patients.

## INTRODUCTION

The study of visual perception in patients with schizophrenia is increasing knowledge about the brain mechanisms that are impaired in this devastating neurocognitive disorder (for a review see Butler et al., 2008) that affects 1% of world population (Lewis and Lieberman, 2000). It is well known that cognitive processes and information processing are seriously affected in schizophrenia and much work has focused on processes like attention, memory or executive functions. However, given the deep knowledge that we have about the visual system in healthy population, there is a growing interest in the study of perceptual processes, in particular, visual perception (Yoon et al., 2009; Chen, 2011; Kim et al., 2011; Robol et al., 2013; Tibber et al., 2013; Yang et al., 2013).

The main findings about visual perception in schizophrenia have shown that there is: (a) reduced contrast sensitivity (Slaghuis, 1998; Kéri et al., 2002; Butler et al., 2005; Kantrowitz et al., 2009; for a review see Skottun and Skoyles, 2007); (b) an altered perception of visual illusions (Uhlhaas and Mishara, 2007; Kantrowitz et al., 2009; for a review see Notredame et al., 2014); (c) less susceptibility to contrast illusions in stimuli surrounded by regions of high contrast (Dakin et al., 2005; Yang et al., 2013; though Barch et al., 2012) failed to replicate this effect; (d) poor stereoacuity (Schechter et al., 2006; Kantrowitz et al., 2009); (e) weak center-surround suppression using motion stimuli (Tadin et al., 2006), although Chen et al. (2008) found the opposite result, surround suppression was abnormally increased in schizophrenia; (f) reduced facilitation to detect a stimulus presented with two collinear flankers with same spatial frequency and orientation located at a particular distance (Must et al., 2004; Kéri et al., 2005); (g) reduced orientation-surround suppression when the task was to discriminate a stimulus surrounded by regions with same spatial frequency and orientation (Yoon et al., 2009, 2010); and (h) a broader orientation tuning (Rokem et al., 2011).

In summary, visual perception in schizophrenia is in general altered and shows poor performance, with reduced suppression, reduced facilitation, poor stereoacuity, and reduced contrast sensitivity. However, this reduced suppression in some cases can enhance performance in tasks where there is contextual modulation, this is, when the perception of a stimulus is affected by a surrounding stimulus (Yang et al., 2013).

Most of the results described here could be related with some neural deficits found in schizophrenia patients like the increased neuronal density (10%) found in the occipital area 17 (Selemon et al., 1995); this neuronal hypertrophy suggests that neuronal connections in the occipital cortex could be affected. There are other factors that can contribute to the differences found between controls and schizophrenic patients. For example, glutamate is the major excitatory neurotransmitter in the brain and one of its receptors [*N*-methyl-D-aspartate (NMDA)] shows a hypofunction in schizophrenia patients (Olney and Farber, 1995; Moghaddam, 2003). NMDA receptors amplify both excitatory and inhibitory visual signals and enhance lateral inhibition in the lateral geniculate nucleus (LGN; Daw et al., 1993). Thus, the hypofunction of the NMDA receptors could potentially explain results where a reduced facilitation was found (Must et al., 2004; Kéri et al., 2005). There is another important neuronal deficit that potentially could explain previous results. The concentration of gamma-aminobutyric acid (GABA) neurotransmitter in visual cortex is about 10% lower in schizophrenia patients (Yoon et al., 2010; see also Wassef et al., 2003 and Rokem et al., 2011). Given that GABA is the main neurotransmitter underlying cortical inhibitory mechanisms, this reduced concentration of GABA neurotransmitter in schizophrenia patients could explain the reduced surround suppression found in schizophrenic patients (Tadin et al., 2006; Yoon et al., 2009). Interestingly, this alteration of the GABAergic inhibition could lead schizophrenia patients to perform better than controls in visual suppression tasks (Tadin et al., 2006).

One example of visual surround suppression is that a grating located in the visual periphery becomes less visible if surrounded by a grating with the same spatial frequency and orientation (Petrov et al., 2005; Lev and Polat, 2011). We hypothesized that if schizophrenia patients have cortical alterations, including hypofunction of NMDA receptors, and reduced concentration of GABA neurotransmitter, that affects lateral inhibitory connections in early visual cortex, then schizophrenia patients should perform better than controls in this visual surround suppression task. This type of orientation-surround suppression has been studied previously with schizophrenia patients using a contrast discrimination task where the participants had to detect a difference in contrast between one segment and other seven segments (Yoon et al., 2009). The main finding was that patients had lower orientation-surround suppression than controls (we will discuss the differences and similarities with this study in the discussion). To test the hypothesis, we measured contrast detection thresholds using a sophisticated Bayesian staircase procedure, using a four-alternative forced-choice (4AFC) task [the recommended task for naïve observers (Jäkel and Wichmann, 2006)] and using a new orientation-surround suppression visual stimulus (Serrano-Pedraza et al., 2012), in 24 healthy subjects and 21 schizophrenia patients. Our results generalize the results found by Yoon et al. (2009) to a detection task and support the hypothesis that inhibitory connections in early visual cortex are impaired in schizophrenia patients.

## MATERIALS AND METHODS

### SUBJECTS

The present cross-sectional study was carried out with 21 patients (we mixed 11 outpatients and 10 inpatients aged from 24 to 58 years, mean age was 39.19 years, SD = 8.99) who met DSM-IV criteria APA (1994) for paranoid schizophrenia (**Table 1**), and 24 age-matched controls (aged from 24 to 58 years, mean age was 39.7 years, SD = 9.71) with no self-reported history of psychiatric illness. All patients were diagnosed using the Structured Clinical Interview for DSM-IV Axis I Disorders (SCID-I) (First et al., 2002) and all of them were taking second-generation antipsychotic medication. Daily doses of medication were converted to chlorpromazine equivalent doses (Woods, 2003; Andreasen et al., 2010). The patient group was recruited from Hospital 12 de Octubre. Clinical status was evaluated using the Spanish version of the positive and negative syndrome scale (PANSS; Kay et al., 1987; Peralta and Cuesta, 1994). Exclusion criteria for patients were: intelligence quotient <70, history of head trauma, and drug or alcohol dependence. All subjects (controls and patients) had normal or corrected-to-normal refraction. The experiments were carried out in a room with a dim light and a chin-rest (UHCOTech HeadSpot, Houston, TX, USA) was used to stabilize the subject’s head and to control the observation distance. The subjects were instructed to maintain fixation on a small cross (0.25° × 0.25°) in the center of the screen before presenting the stimuli. Experimental procedures were approved by the Ethics Committee of Complutense University of Madrid, Spain. A written informed consent was obtained from all participants prior to their inclusion in the study.

**Table 1:** Participants demographics and clinical data.

	Patients (*n* = 21)	Controls (*n* = 24)	
Age	39.19 (24–58; SD 8.99)	39.7 (24–58; SD 9.71)	*t*-test, *p*= 0.854
Gender	19M 2F	10M 14F	
Education (Years)	9.25 (SD 1.48)	16.4 (SD 4.54)	*t*-test, *p*< 0.01
Illness duration (years)	19.9 (SD 10.74)	–	
PANSS-P	14.90 (SD 6.78)	–	
PANSS-N	20.61 (SD 6.41)	–	
PANSS-GP	33.61 (SD 8.09)	–	
CPZ equivalent dose*	638.189 (SD 408.28)	–	

Mean and SD, standard deviation.

*Chlorpromazine equivalent dose (mg/day).

### EQUIPMENT

The images were presented in a γ-corrected 23-inch LG monitor (D2342P) under the control of a Mac Pro running Matlab (MathWorks, Natick, MA, USA) using the Psychophysics Toolbox extensions (Brainard, 1997; Pelli, 1997; Kleiner et al., 2007)^1^ and using the Visual stimulator DataPixx Lite (VPixx Technologies Inc., Canada^2^) that gave us a measured 10-bits of gray scale resolution. The responses were recorded using a response box (ResponsePixx Handheld, VPixx Technologies Inc., Canada). We used two identical setups (same model of monitor, visual stimulator, and response box) for testing all participants. All patients and 17% of controls were tested with one setup and the rest of control subjects with the other setup. The monitors were carefully gamma corrected using a PR-650 SpectraScan Colorimeter (Photo Research, Inc., USA^3^), had a resolution of 1920 × 1080 pixels (horizontal × vertical) subtending a visual angle of 54.04 × 32.01° (horizontal × vertical) with vertical frame rate of 60 Hz, a mean luminance of 37.9 cd/m^2^, and were observed binocularly (in a dimly lit room) from a distance of 50 cm. Given that patients would be tested with a different set-up (although the equipment was identical), we tested both set-ups with five control subjects with experience in psychophysical experiments and that did not participate in the main experiment. They performed the same experimental conditions described in *Procedure* section [no-surround (NS), orthogonal, and parallel]. Each subject ran the experiment three times in both set-ups. We compared the means of the contrast thresholds of these five subjects between set-ups using a two-tailed *t*-test (α = 0.05) and we found no significant differences between set-ups in the three conditions: NS [*t*(4) = 1.936, *p* = 0.125], orthogonal-surround [OS; *t*(4) = 0.452, *p* = 0.675], and parallel-surround [PS; *t*(4) = 0.352, *p* = 0.742].

### STIMULI

**FIGURE 1:**
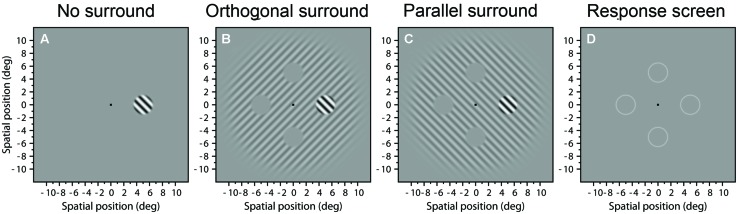
**Example stimuli.** Images **(A–C)** show examples of stimuli presented in the three experimental conditions: **(A)** No surround; **(B)** Orthogonal surround; and **(C)** Parallel surround. **(D)** Response screen with four circles signaling the possible positions where the target appeared.

The stimuli were based on the stimuli used in Serrano-Pedraza et al. (2012) and had some similarities from stimuli used by; Cannon and Fullenkamp (1991), Petrov et al. (2005), and Yoon et al. (2010). The target was a grating of spatial frequency of 1 c/deg with a 3°-Butterworth spatial window of order 10 [see González and Wintz (1987, p. 179, 181) and Sierra-Vazquez et al. (2006), a formal definition can be seen in their appendix A] that appeared randomly in one of four possible positions at 5° eccentricity (see **Figure 1D**) We chose this eccentricity because surround suppression is stronger in periphery than in fovea, and reaches a plateau at eccentricities greater than 4° (Petrov et al., 2005).

We tested three experimental conditions where the target could appear with no-surround (**Figure 1A**), embedded within a surround grating with orthogonal orientation (**Figure 1B**), or with the same (parallel) orientation (**Figure 1C**). The surround gratings had a fixed Michelson contrast of 0.25 and a 20°-Butterworth window of order 10. The surround grating had four “holes,” i.e., gray locations centered at 5° eccentricity at the four possible target locations. The holes were 3.05° in diameter, i.e., there was a 0.05° gap between the edges of the target and the surround. The orientation of the target and surround was randomly ±45°. The phase of the target and the surround was the same but randomized each trial between 0 and 2π. Olzak and Laurinen (1999) found that stronger suppression occurs when targets and surrounds with same orientation are in phase, although Petrov and McKee (2006) found that surround suppression is not affected by the phase of the surround. The remainder of the screen was at the mean luminance.

We used a Bayesian adaptive staircase to measure contrast detection thresholds in a spatial 4AFC detection task. This is the recommended task for naïve observers compared with 2AFC or 8AFC (Jäkel and Wichmann, 2006).

### PROCEDURE

In each trial, target and surround (**Figures 1A–C**) appeared contrast-modulated in time by a Gaussian temporal function with mean 500 ms and σ_t_ = 100 ms (duration of 200 ms, 2σ_t_), truncated to give an overall duration of 1000 ms. In the center of the stimulus, during the presentation, appeared a small thin line turning clockwise with the objective of attracting the attention of the subject to the center of the screen. The particular contrast of the target was controlled by an adaptive staircase procedure. After the stimulus presentation, a fixation-cross was displayed at the center of the screen surrounded by four circles signaling the possible positions where the target appeared (see **Figure 1D**). The subject’s task was to indicate the position of the target by pressing button of the response box. In the first trial the subject had to press any button to start. A new trial was initiated after the observer’s response, thus the experiment proceeded at a pace determined by the observer.

Contrast detection threshold was defined as the minimum Michelson contrast that is needed in order to detect the correct target image, resulting in a performance of 62% correct. Thus, a low contrast threshold means that the subject has high sensitivity (sensitivity is defined as the inverse of the threshold). Contrast thresholds were measured using adaptive Bayesian staircases (Treutwein, 1995) in a forced-choice detection task. The characteristics of the Bayesian staircases were: (1) the prior probability density function was uniform (Emerson, 1986) with a starting contrast of 0.99; (2) we used the logistic function adapted from (García-Pérez, 1998; see his appendix A) as the model likelihood function with a spread value of 1, delta parameter equal to 0.01, a lapse rate of 0.015, and a guess rate of 0.25; (3) the value of the target contrast in each trial was obtained from the mean of the posterior probability distribution (King-Smith et al., 1994); (4) the staircase stopped after 30 trials (Anderson, 2003); and (5) the final threshold was estimated from the mean of the final probability density function. The three experimental conditions (NS, orthogonal, and parallel) were tested in the same experimental session interleaving randomly the conditions across trials. Practice sessions were performed previous to the experiment.

### STATISTICAL ANALYSES

Our dependent variable was the contrast threshold needed to detect the target correctly 62% of the presentations using a 4AFC method. We had three conditions: NS, OS, and PS. In order to compare the contrast thresholds of control group and patients group for each condition, first, we calculated the mean of each group in each condition; second we computed the *p*-values using the Smith-Welch-Satterthwaite test (Ruxton, 2006) or unequal variance *t*-test if the Bartlett’s test for equality of variances was significant, and a Student’s *t*-test in other case (two-tailed, α = 0.05). We also analyzed the interaction between condition and group using a multi-sample repeated measures analysis of variance (ANOVA) with two factors. To study the evolution of contrast thresholds as a function age we used simple linear regression, we computed the coefficient of determination *R*^2^, the *p*-value for the slope. We will show the correlation coefficient *r* when *p* < 0.05.

## RESULTS

Forty-five participants (24 controls and 21 patients with schizophrenia) took part in a visual psychophysics experiment. Contrast detection thresholds were obtained in three conditions (NS, OS, and PS).

**FIGURE 2:**
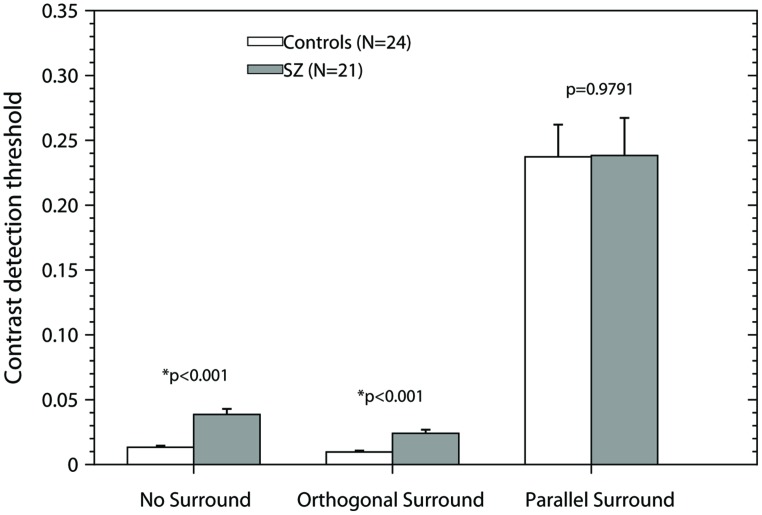
**Contrast detection thresholds as a function of the experimental condition.** Bars represent the mean + SEM. Gray bars, data of patients; white bars, data of controls. *p*-values were obtained using the Smith-Welch-Satterthwaite test or unequal variance *t*-test when Bartlett’s test was significant and a *t*-test in other case (two-tailed, α = 0.05). Asterisks (*) correspond to significant differences between the means of the contrast detection thresholds.

**Figure 2** shows the results of the experiment. The bars represent the mean + SEM for each condition and group. White bars represent the thresholds of the control group; black bars represent the results of the patients group. In both the NS and OS condition, contrast thresholds are significantly lower for the control group than for patients [unequal variance two-tailed *t*-test, NS: *t*(23.14) = 5.64, *p* < 0.001; OS: *t*(25.77) = 4.84, *p* < 0.001], that is the control group has higher sensitivity than the patient group. However, no significant differences were found for the PS condition [two-tailed Student’s *t*-test, *t*(43) = 0.026, *p* = 0.9791]. Both groups had higher thresholds in the suppressive condition (PS) that the NS and OS conditions replicating previous results (Serrano-Pedraza et al., 2012). The elevated thresholds in the PS condition represent the suppressive effect of the surround. Repeated measures ANOVA with two factors, condition (NS, OS, and PS) and group (Controls and Patients) demonstrated a significant effect of condition (*F*_2,86_ = 137.41, *p* < 0.001), no effect of group (*F*_1,43_ = 1.02, *p* = 0.3192), and no significant condition × group interaction (*F*_2,86_ = 0.32, *p* = 0.7263), thus, the effect of the condition (NS, OS, and PS) on performance is the same independent of the group.

Given that our patient group is predominately male (19M, 2F) and also different from the control group (10M, 14F) we analyzed if there were sex differences in this experiment using the control group. We found no significant differences between males and females in the three experimental conditions: two-tailed *t*-test, NS [*t*(22) = 0.324, *p* = 0.748], OS [*t*(22) = 0.07, *p* = 0.943], and PS [*t*(22) = 1.386, *p* = 0.179]. Comparisons of males in both groups (controls, *n* = 10; patients, *n* = 19) showed similar results: unequal variance two-tailed *t*-test, NS: *t*(25.47) = 4.819, *p* < 0.001; OS: *t*(24.1) = 4.393, *p* < 0.001; two-tailed *t*-test: PS: *t*(27) = 0.738, *p* = 0.466.

**FIGURE 3:**
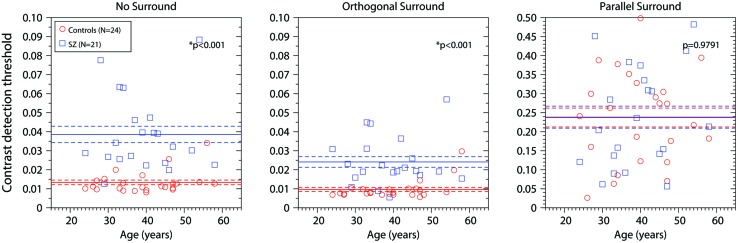
**Contrast detection thresholds as a function of age (years) for the three conditions (No-Surround, Orthogonal Surround, and Parallel Surround).** Blue squares, contrast detection thresholds for patients; red circles, contrast detection thresholds for control group. The blue horizontal line represents the mean ± SEM for patients; the red horizontal line represents the mean ± SEM for the control group. *p*-values are the same as in **Figure 2**. Asterisks (*) correspond to significant differences between the means of the contrast detection thresholds.

We also examined the effect of age. **Figure 3** shows the results for each participant as a function of age (years). Blue squares represent the data of the patient group; red dots represent the data of the control group. For each group, the horizontal line corresponds to the mean value and the dotted line ±SEM (data represented in **Figure 2**, white bars). Each panel shows the results for one experimental condition. In the control group, for the OS condition only, there was a small but significant increase in contrast threshold with age (*r* = 0.559, *R*^2^ = 0.313, *p* = 0.004). This was driven mainly by the two oldest controls, and was not significant if they were removed (*R*^2^ = 0.043, *p* = 0.3502). There was no significant dependence on age for controls in the other two conditions, or for patients in any condition. Thus, in general thresholds did not depend strongly on age, at least over the range included in our sample (24–58 years).

The performance of individual participants was correlated across the three different conditions. When PS thresholds are plotted against NS thresholds, there is a small correlation for both groups, only significant for patients (controls: *R*^2^ = 0.132, *p* = 0.08; patients: *r* = 0.469, *R*^2^ = 0.22, *p* = 0.031). That is, the higher the contrast threshold in the NS condition, the higher the contrast thresholds in the PS condition. There was a significant difference in the slope of the regression line between the two groups (slope controls = 7.46, slope patients = 3.15, *p* = 0.027). We also represented the OS thresholds as a function of the NS thresholds and found significant correlations for both groups (controls: *r* = 0.397, *R*^2^ = 0.1576, *p* = 0.05; patients: *r* = 0.674, *R*^2^ = 0.455, *p* < 0.001). Thus, in order to quantify the visual suppression in both groups we computed a Surround Suppression Index (see Tadin et al., 2006). This was defined as the log ratio between the contrast thresholds of the PS condition and the NS condition. We also calculated this log ratio for the OS condition. By normalizing each participant against their own performance in a different condition, this helps to remove the effect of inter-subject differences and more clearly reveal the effect of surround.

**FIGURE 4:**
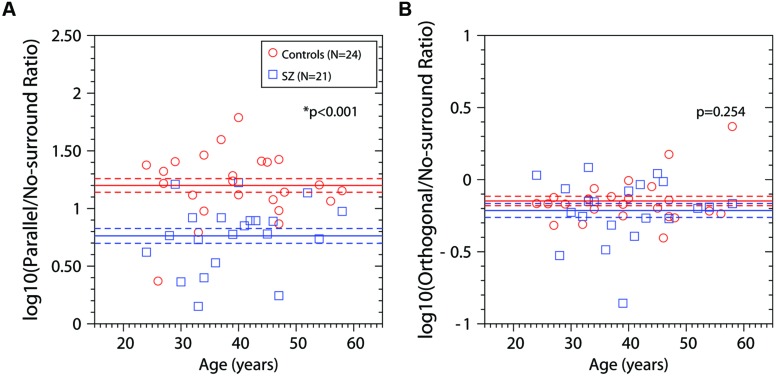
**Log ratios of contrast detection thresholds as a function of age (years). (A)** panel shows the log ratios between the contrast detection threshold in the condition Parallel Surround and the condition No-Surround. **(B)** panel shows the log ratios between the contrast detection threshold in the condition Orthogonal Surround and the condition No-Surround. Blue squares, ratios for patients; red circles, ratios for control group. The blue horizontal line represents the mean ± SEM for patients; the red horizontal line represents the mean ± SEM for the control group. *p*-values were obtained using two-tailed *t*-test. Asterisks (*) correspond to significant differences between the means of the log ratios.

**Figure 4** shows the log ratios for each participant and for PS and OS conditions. Blue squares correspond to the data of the patients group; red dots represent the data of the control group. The red and blue horizontal lines represent the mean ±SEM of the log ratios for control and patients group respectively. We compared the mean of the ratios for both groups. Statistical analysis showed a significant difference between groups for the PS/NS log ratios [two-tailed *t*-test, *t*(43) = 5.034, *p* < 0.001], with the suppression ratios being higher for the control group. We found no difference when comparing OS/NS log ratios between controls and patients [two-tailed *t*-test, *t*(43) = 1.156, *p* = 0.254]. Note that mean values of OS/NS ratios are lower than 0, indicating facilitation, for both groups, thus this ratio could be used as a facilitation index. Repeated measures ANOVA with two factors, condition (PS/NS log ratios, and OS/NS log ratios) and group (Controls and Patients) demonstrated a significant effect of condition (*F*_1,43_ = 531.38, *p* < 0.001), significant effect of group (*F*_1,43_ = 22.69, *p* < 0.001), and a significant condition × group interaction (*F*_1,43_ = 13.25, *p* < 0.001). Regression analysis for ratios as a function of age showed that there is no significant dependence on age for either group (control or patients) or ratio (parallel or orthogonal; Controls: parallel ratio, *R*^2^ = 0.001, *p* = 0.882; orthogonal ratio, *R*^2^ = 0.075, *p* = 0.192; Patients: parallel-ratio, *R*^2^ = 0.06, *p* = 0.278; orthogonal ratio, *R*^2^ = 0.0001, *p* = 0.892). Regression analysis for PS/NS log ratios as a function of orthogonal/no-surround log ratios showed no significant dependence either (*R*^2^ = 0.011, *p* = 0.646). Finally, regression analysis for suppression ratios (PS/NS log ratios) as a function of no-surround contrast thresholds showed no significant correlation neither for controls (*R*^2^ = 0.019, *p* = 0.513) nor patients (*R*^2^ = 0.117, *p* = 0.127).

**FIGURE 5:**
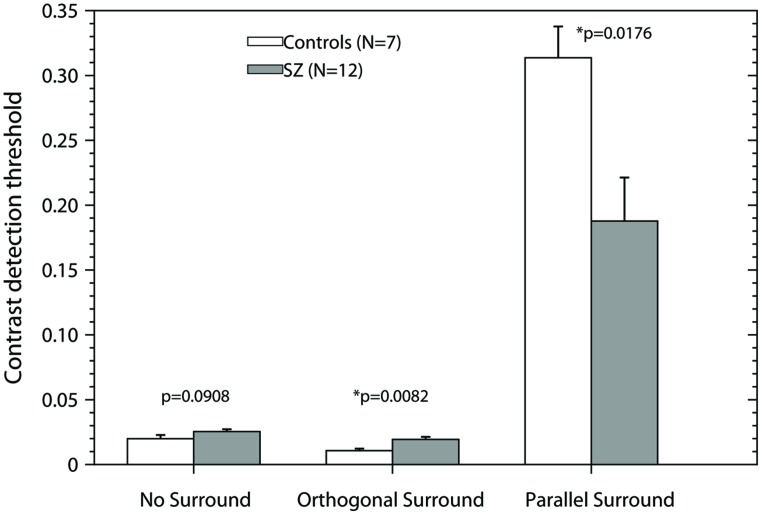
**Contrast detection thresholds as a function of the experimental condition.** For this analysis, we selected the controls in which contrast detection thresholds in the condition No-Surround were higher that the mean of the group for that condition, and the patients, in which contrast detection thresholds for No-Surround condition, were lower that the mean of the group. Bars represent the mean + SEM. Gray bars, data of patients; white bars, data of controls. *p*-values were obtained using two-tailed *t*-test (α = 0.05). Asterisks (*) correspond to significant differences between the means of the contrast detection thresholds. Note that in the condition Parallel Surround the patients have significant lower thresholds than controls.

In **Figure 4** we have shown that suppression ratios for PS condition were significant higher for controls than for patients, indicating stronger suppression in controls. However, as **Figure 2** shows, in fact the thresholds for the PS condition were similar between groups. The difference in suppression ratios is driven by the difference in thresholds for the NS condition, which were significantly lower for controls than for patients. We asked whether the difference in suppression ratios persists and whether there are differences in contrast thresholds in the parallel condition when controlling for differences in contrast thresholds in the NS condition. We chose a subset of controls in which contrast thresholds were higher than the group mean (*N* = 7, mean contrast threshold = 0.0199), and a subset of patients which contrast thresholds were lower than the group mean (*N* = 12, mean contrast threshold = 0.025; note therefore that this subset of controls and patients are not selected randomly and they are not representative of the population of controls and patients). **Figure 5** shows the results for these subsets. The bars represent the mean + SEM of the contrast thresholds. In the NS condition, by design, there were of course no significant differences between the selected patients and controls [two-tailed *t*-test, *t*(17) = 1.79, *p* = 0.0908]. However, significant differences persisted in both the other conditions [two-tailed *t*-test: OS, *t*(17) = 2.988, *p* = 0.0082; and PS, *t*(17) = 2.626, *p* = 0.0176]. Interestingly, the data show that patients with schizophrenia had significantly lower thresholds than controls in the PS condition, so they perceived the visual stimulus better than controls. Repeated measures ANOVA for this subset of controls and patients, with two factors, condition (PS/NS log ratios, and OS/NS log ratios) and group (Controls and Patients), showed a significant effect of condition (*F*_1,17_ = 369.33, *p* < 0.001), non-significant effect of group (*F*_1,17_ = 2.75, *p* = 0.115), and a significant condition × group interaction (*F*_1,17_ = 19.91, *p* < 0.001). *Post hoc t*-test using Bonferroni correction revealed that suppression ratios (PS/NS) were significantly lower for patients than for controls (*p* = 0.021); and no differences were found for facilitation ratios (OS/NS) between controls and patients (*p* = 0.216).

Finally, we performed a Regression analysis for contrast thresholds of 21 patients as a function of their clinical data (see **Table 2**) and for PS/NS and OS/NS log ratios and clinical data (see **Table 3**). Results show that there is no significant dependence between our measurements (contrast thresholds and ratios) and clinical symptoms and medication.

**Table 2:** Regression analysis for contrast detection thresholds as a function of clinical symptoms and medication (*n* = 21 patients).

	No-surround	Orthogonal-surround	Parallel-surround
PANSS-P	*R*^2^ = 0.000, *p* = 0.99	*R*^2^ = 0.045, *p* = 0.356	*R*^2^ = 0.088, *p* = 0.19
PANSS-N	*R*^2^ = 0.000, *p* = 0.958	*R*^2^ = 0.0125, *p* = 0.628	*R*^2^= 0.003, *p* = 0.806
PANSS-GP	*R*^2^= 0.002, *p* = 0.821	*R*^2^ = 0.049, *p* = 0.334	*R*^2^ = 0.084, *p* = 0.20
CPZ*	*R*^2^ = 0.003, *p* = 0.799	*R*^2^ = 0.009, *p* = 0.682	*R*^2^ = 0.082, *p* = 0.209

Table shows the coefficient of determination *R*^2^ and the *p*-value for the slope.

*Chlorpromazine equivalent dose (mg/day).

**Table 3:** Regression analysis for PS/NS and OS/NS log ratios as a function of clinical symptoms and medication (*n* = 21 patients).

	PS/NS Log ratio	OS/NS Log ratio
PANSS-P	*R*^2^ = 0.063, *p* = 0.272	*R*^2^ = 0.038, *p* = 0.392
PANSS-N	*R*^2^ = 0.003, *p* = 0.791	*R*^2^ = 0.001, *p* = 0.865
PANSS-GP	*R*^2^ = 0.137, *p* = 0.103	*R*^2^ = 0.071, *p* = 0.245
CPZ*	*R*^2^ = 0.111, *p* = 0.139	*R*^2^ = 0.000, *p* = 0.973

Table shows the coefficient of determination *R*^2^ and the *p*-value for the slope.

*Chlorpromazine equivalent dose (mg/day).

## DISCUSSION

We presented contrast detection thresholds of 24 healthy subjects and 21 schizophrenia patients when performing a task designed to measure visual suppression.

We found that a grating located in periphery without surround, or with an OS, was detected with lower contrast thresholds by the control group, therefore, the schizophrenia patients showed a reduced contrast sensitivity (defined as the inverse of contrast threshold). The differences found when the target was presented without surround replicates previous results where it has been found that schizophrenia patients have a general reduction of contrast sensitivity (see a review in Skottun and Skoyles, 2007).

Consistent with previous reports (Serrano-Pedraza et al., 2012), patients and controls showed a facilitatory effect, that is, slightly lower thresholds in the OS condition than in the NS condition, i.e., an OS facilitated the detection of the target. Reflecting their lowered contrast sensitivity, patients showed higher thresholds than controls in both the OS and NS conditions, but there was no difference in the facilitation index between controls and patients, defined as the log-ratio of thresholds in orthogonal and NS conditions (OS/NS log ratio, **Figure 4**, right panel). This facilitatory effect appears to occur only at low contrasts, since Yoon et al. (2009) found no facilitation in either patients or controls on a discrimination task requiring subjects to detect changes from a baseline contrast of 75%; in fact, OS discrimination thresholds were higher than NS discrimination thresholds, implying suppression. Like us, they did not find a difference between schizophrenia patients and controls in the OS/NS ratio. Thus, both we and Yoon et al. (2009) find that an orthogonally oriented surround has similar effects in both schizophrenic patients and controls, at both high and threshold contrasts.

Kéri et al. (2005) used a detection task with a different geometry, in which subjects were asked to detect a Gabor patch presented either in isolation (analogous to our NS condition), or in between two flanking Gabors of the same orientation but 40% contrast. When the flankers were close to the target, contrast thresholds were increased, presumably by the same suppressive mechanism as in our PS condition. However, when flankers were placed further away from the target, they had a facilitative effect in control subjects: contrast thresholds fell below the value needed to detect an isolated patch. This facilitation was not observed in schizophrenic patients. This may suggest that facilitation by distant patches of parallel orientation, as in Kéri et al. (2005), reflects different neuronal mechanisms than facilitation by an OS. Alternatively, spatial frequency differences may contribute, Kéri et al. (2005) used patches of 6.7 c/deg, much higher than our 1 c/deg.

When the surround orientation was parallel to the target (suppression condition), we found higher contrast thresholds than in the NS and OS conditions, and no significant differences between controls and schizophrenia patients. To quantify the strength of the suppression of our participants, we defined the suppression index as the log-ratio of the thresholds in the PS and NS conditions. We found that the suppression indices were significantly lower for schizophrenia patients than for controls. Physiology studies in cats (Blakemore and Tobin, 1972; DeAngelis et al., 1994) macaques (Cavanaugh et al., 2002; Webb et al., 2005), and mice (Self et al., 2014) have shown that the stimulation of a visual neuron outside of its classical receptive field can produce strong suppression of the neuron’s response. This suppression is strong when both, the stimulus presented in the surround and the stimulus presented in the center of the neuron’s receptive field, have the same spatial frequency and orientation. Although the neural mechanisms of surround suppression are not completely understood, three possible sources of surround suppression have been proposed: (a) thalamic source with local inhibitory connections within the LGN, (b) long-range lateral connections within V1; and (c) feedback connections to V1 from extrastriate cortex (see Angelucci et al., 2002; Angelucci and Bressloff, 2006; Smith, 2006). Suppression in the LGN is non-orientation-tuned (Solomon et al., 2002; Bonin et al., 2005), thus, given that in our task, contrast thresholds depended on the orientation of the surround (Petrov et al., 2005; Serrano-Pedraza et al., 2012) we can assume that suppression in the LGN cannot account for the effect of orientation that we have found in this task. On the other hand, it has been shown that lateral connections within V1 can account for surround diameters up to 5.5° at 5° foveal eccentricity (near surround, 4 times the size of V1 minimum response field), and feedback connections to V1 can account for surround diameters higher than 13° (far surround, 23 times the size of V1 minimum response field; Angelucci et al., 2002). Our targets had a diameter of 3° and were presented at 5° eccentricity and the surround extended up to 10° around the target, so both lateral and feedback inhibitory connections could potentially contribute. In either case, the reduced suppression found in our patients supports the hypothesis that inhibitory connections in visual cortex are impaired in schizophrenia.

As noted, patients had lower suppression indices because they had higher thresholds than controls in the NS condition, not because they were better than controls in the PS conditions. The poorer performance of patients in the NS condition may reflect impaired processing in visual periphery in schizophrenia (Kraehenmann et al., 2012), as well as attentional deficits (Barch et al., 2012). Medication and clinical symptoms could also have an effect on contrast thresholds in all conditions; however, we did not find any significant correlation between Chlorpromazine equivalent dose or PANSS scores with contrast thresholds, suppression ratios, and facilitation ratios. We also compared patients to a subgroup of controls, matched for contrast thresholds in the NS condition. In the facilitation (OS) condition, these matched controls had significantly lower contrast thresholds than the patients, however, schizophrenia patients showed lower contrast thresholds in the suppression (PS) condition. The overall picture is that schizophrenia patients generally perform poorly on peripheral contrast detection tasks relative to controls, but they perform *relatively* much better in the PS condition. We interpret this as reduced visual surround suppression, probably related to a reduced concentration of GABA neurotransmitter (Yoon et al., 2010).

Recent studies, using different stimulus configurations, have also found evidence for weaker visual surround suppression in schizophrenia. For example Tibber et al. (2013) showed that schizophrenia patients have weaker surround suppression for stimuli defined by contrast or size but not for those defined by luminance or orientation. These authors concluded that the reduced surround suppression could not be explained by attention deficits or medication. Yang et al. (2013) also found weakened contextual modulations of contrast that made patients with schizophrenia perform more accurately than controls.

Our study has most similarities with Yoon et al. (2009) but it also has important differences. The major difference is that they used a contrast discrimination task and we used a contrast detection task. The behavior of visual system in contrast processing is different at threshold than at suprathreshold values (e.g., Tiippana and Näsänen, 1999). Thus, results obtained with suprathreshold values are difficult to generalize to threshold conditions. There are other differences like the stimulus used (spatial configuration, presentation time, contrast of surround) and the 4AFC method (recommended for naïve observers, Jäkel and Wichmann, 2006). Interestingly, despite these differences, we reach similar conclusions. This confirmation is important given the conflicting results in this area, reviewed briefly in the Introduction.

Taking all results together, our study provides further evidence of abnormal visual processing in schizophrenia, possibly due to impaired inhibitory connections in early visual cortex.

## AUTHOR CONTRIBUTIONS

Ignacio Serrano-Pedraza designed the study, performed the experiments, analyzed the data, and wrote the manuscript. Verónica Romero-Ferreiro, performed the experiments and contributed with the statistical analysis. Jenny C. A. Read, helped with the design of the study and commented on the manuscript. Teresa Diéguez-Risco, performed the experiments and helped with the statistical analysis. Alexandra Bagney, Montserrat Caballero-González, and Javier Rodríguez-Torresano, selected the patient sample and evaluated the patients. Roberto Rodriguez-Jimenez, supervised the study and contributed with the interpretation of the results. All authors contributed to and have approved the final manuscript.

## Conflict of Interest Statement

The authors declare that the research was conducted in the absence of any commercial or financial relationships that could be construed as a potential conflict of interest.

## Footnotes

1 www.psychtoolbox.org

2 http://www.vpixx.com

3 http://www.photoresearch.com

## References

[B1] Anderson A. J. (2003). Utility of a dynamic termination criterion in the ZEST adaptive threshold method. *Vision Res.* 43 165–170 10.1016/S0042-6989(02)00396-6 12536138

[B2] Andreasen N. C. Pressler M. Nopoulos P. Miller D. Ho B. C. (2010). Antipsychotic dose equivalents and dose-years: a standardized method for comparing exposure to different drugs. *Biol. Psychiatry* 67 255–262 10.1016/j.biopsych.2009.08.040 19897178 PMC3677042

[B3] Angelucci A. Bressloff P. (2006). Contribution of feedforward, lateral and feedback connections to the classical receptive field center and extraclassical receptive field surround of primate V1 neurons. *Prog. Brain Res.* 154 93–120 10.1016/S0079-6123(06)54005-1 17010705

[B4] Angelucci A. Levitt J. Lund J. (2002). Anatomical origins of the classical receptive field and modulatory surround field of single neurons in macaque visual cortical area V1. *Prog. Brain Res.* 136 373–388 10.1016/S0079-6123(02)36031-X 12143395

[B5] APA. (1994). *Diagnostic and Statistical Manual of Mental Disorders: DSM-IV* 4th Edn. Washington, DC: American Psychiatric Association.

[B6] Barch D. Carter C. Dakin S. Gold J. Luck S. Macdonald A. III (2012). The clinical translation of a measure of gain control: the contrast-contrast effect task. *Schizophr. Bull.* 38 135–143 10.1093/schbul/sbr154 22101963 PMC3245599

[B7] Blakemore C. Tobin E. A. (1972). Lateral inhibition between orientation detectors in the cat’s visual cortex. *Exp. Brain Res.* 15 439–440 10.1007/BF00234129 5079475

[B8] Bonin V. Mante V. Carandini M. (2005). The suppressive field of neurons in lateral geniculate nucleus. *J. Neurosci.* 25 10844–10856 10.1523/JNEUROSCI.3562-05.2005 16306397 PMC6725877

[B9] Brainard D. H. (1997). The psychophysics toolbox. *Spat. Vis.* 10 433–436 10.1163/156856897X00357 9176952

[B10] Butler P. Silverstein S. M. Dakin S. (2008). Visual perception and its impairment in schizophrenia. *Biol. Psychiatry* 64 40–47 10.1016/j.biopsych.2008.03.023 18549875 PMC2435292

[B11] Butler P. D. Zemon V. Schechter I. Saperstein A. M. Hoptman M. J. Lim K. O. (2005). Early-stage visual processing and cortical amplification deficits in schizophrenia. *Arch. Gen. Psychiatry* 62 495–504 10.1001/archpsyc.62.5.495 15867102 PMC1298183

[B12] Cannon M. W. Fullenkamp S. C. (1991). Spatial interactions in apparent contrast: inhibitory effects among grating patterns of different spatial frequencies, spatial positions and orientations. *Vision Res.* 31 1985–1998 10.1016/0042-6989(91)90193-9 1771782

[B13] Cavanaugh J. R. Bair W. Movshon J. A. (2002). Selectivity and spatial distribution of signals from the receptive field surround in macaque V1 neurons. *J. Neurophysiol.* 88 2547–2556 10.1152/jn.00693.2001 12424293

[B14] Chen Y. (2011). Abnormal visual motion processing in schizophrenia: a review of research progress. *Schizophr. Bull.* 37 709–715 10.1093/schbul/sbr020 21436317 PMC3122297

[B15] Chen Y. Norton D. Ongur D. (2008). Altered center-surround motion inhibition in schizophrenia. *Biol. Psychiatry* 64 74–77 10.1016/j.biopsych.2007.11.017 18206855 PMC2483430

[B16] Dakin S. C. Carlin P. Hemsley D. (2005). Weak suppression of visual context in chronic schizophrenia. *Curr. Biol.* 15 822–824 10.1016/j.cub.2005.10.015 16243017

[B17] Daw N. W. Stein P. S. G. Fox K. (1993). The role of NMDA receptors in information processing. *Annu. Rev. Neurosci.* 16 207–222 10.1146/annurev.ne.16.030193.001231 8460891

[B18] DeAngelis G. C. Freeman R. D. Ohzawa I. (1994). Length and width tuning of neurons in the cat’s primary visual cortex. *J. Neurophysiol.* 71 347–374. 8158236 10.1152/jn.1994.71.1.347

[B19] Emerson P. L. (1986). Observations on maximum-likelihood and Bayesian methods of forced-choice sequential threshold estimation. *Percept. Psychophys.* 39 151–153 10.3758/BF03211498 3725540

[B20] First M. G. Spitzer R. L. Gibbon M. Williams J. B. (2002). *Structured Clinical Interview for DSM-IV-TR Axis I Disorders, Research Version, Patient Edition. (SCID-I/P).* New York: Biometrics Research, New York State Psychiatric Institute.

[B21] García-Pérez M. A. (1998). Forced-choice staircases with fixed steps sizes: asymptotic and small-sample properties. *Vision Res.* 38 1861–1881 10.1016/S0042-6989(97)00340-4 9797963

[B22] González R. C. Wintz P. (1987). *Digital Image Processing* 2nd Edn. Reading, MA: Addison-Wesley.

[B23] Jäkel F. Wichmann F. A. (2006). Spatial four-alternative forced-choiced method is the preferred psychophysical method for naïve observers. *J. Vis.* 6 1307–1322 10.1167/6.11.13 17209737

[B24] Kantrowitz J. T. Butler P. D. Schecter I. Silipo G. Javitt D. C. (2009). Seeing the world dimly: the impact of early visual deficits on visual experience in schizophrenia. *Schizophr. Bull.* 35 1085–1094 10.1093/schbul/sbp100 19793795 PMC2762627

[B25] Kay S. R. Fishbein A. Olper L. A. (1987). The positive and negative syndrome scales. *Schizophr. Bull.* 13 261–276 10.1093/schbul/13.2.261 3616518

[B26] Kéri S. Antal A. Szekeres G. Benedek G. Janka Z. (2002). Spatiotemporal visual processing in schizophrenia. *J. Neuropsychiatry Clin. Neurosci.* 14 190–196 10.1176/appi.neuropsych.14.2.190 11983794

[B27] Kéri S. Kelemen O. Benedek G. Janka Z. (2005). Lateral interactions in the visual cortex of patients with schizophrenia and bipolar disorder. *Psychol. Med.* 35 1043–1051 10.1017/S0033291705004381 16045070

[B28] Kim J. Park S. Blake R. (2011). Perception of biological motion in schizophrenia and healthy individuals: a behavioral and fMRI study. *PLoS ONE* 6:e19971 10.1371/journal.pone.0019971 PMC3098848 21625492

[B29] King-Smith P. E. Grigsby S. S. Vingrys A. J. Benes S. C. Supowit A. (1994). Efficient and unbiased modifications of the QUEST threshold method: theory, simulations, experimental evaluation and practical implementation. *Vision Res.* 34 885–912 10.1016/0042-6989(94)90039-6 8160402

[B30] Kleiner M. Brainard D. H. Pelli D. G. (2007). What’s new in Psychotoolbox-3? *Perception* 36 (ECVP Abstract Supplement).

[B31] Kraehenmann R. Vollenweider F. X. Seifritz E. Kometer M. (2012). Crowding Deficits in the Visual Periphery of Schizophrenia Patients. *PLoS ONE* 7:e45884 10.1371/journal.pone.0045884 PMC3458825 23049884

[B32] Lev M. Polat U. (2011). Collinear facilitation and suppression at the periphery. *Vision Res.* 52 2488–2498 10.1016/j.visres.2011.10.008 22037360

[B33] Lewis D. A. Lieberman J. A. (2000). Catching up on schizophrenia: natural history and neurobiology. *Neuron* 28 325–334 10.1016/S0896-6273(00)00111-2 11144342

[B34] Moghaddam B. (2003). Bringing order to the glutamate chaos in schizophrenia. *Neuron* 28 325–334 10.1016/S0896-6273(03)00757-8 14659087

[B35] Must A. Janka Z. Benedek G. Keri S. (2004). Reduced facilitation effect of collinear flankers on contrast detection reveals impaired lateral connectivity in the visual cortex of schizophrenia patients. *Neurosci. Lett.* 357 131–134 10.1016/j.neulet.2003.12.046 15036592

[B36] Notredame C.-E. Pins D. Deneve S. Jardri R. (2014). What visual illusions teach us about schizophrenia. *Front. Integr. Neurosci.* 8:63 10.3389/fnint.2014.00063 PMC4130106 25161614

[B37] Olney J. Farber N. (1995). Glutamate receptor dysfunction and schizophrenia. *Arch. Gen. Psychiatry* 52 998–1007 10.1001/archpsyc.1995.03950240016004 7492260

[B38] Olzak L. A. Laurinen P. I. (1999). Multiple gain control processes in contrast-contrast phenomena. *Vision Res.* 39 3983–3987 10.1016/S0042-6989(99)00131-5 10748930

[B39] Pelli D. G. (1997). The VideoToolbox software for visual psychophysics: transforming numbers into movies. *Spat. Vis.* 10 437–442 10.1163/156856897X00366 9176953

[B40] Peralta V. Cuesta M. J. (1994). Validación de la escala de los síndromes positivo y negativo (PANSS) en una muestra de esquizofrénicos españoles. *Actas Luso-Esp Neurol. Psiquiatr.* 22 171–177. 7810373

[B41] Petrov Y. Carandini M. Mckee S. (2005). Two distinct mechanisms of suppression in human vision. *J. Neurosci.* 25 8704–8707 10.1523/JNEUROSCI.2871-05.2005 16177039 PMC1472809

[B42] Petrov Y. McKee S. P. (2006). The effect of spatial configuration on surround suppression of contrast sensitivity. *J. Vis.* 9 224–238. 16643092 10.1167/6.3.4 PMC1472811

[B43] Robol V. Tibber M. Anderson E. Bobin T. Carlin P. Shergill S. S. (2013). Reduced crowding and poor contour detection in schizophrenia are consistent with weak surround inhibition. *PLoS ONE* 8:e60951 10.1371/journal.pone.0060951 PMC3621669 23585865

[B44] Rokem A. Yoon J. H. Ooms R. E. Maddock R. J. Minzenberg M. Silver M. A. (2011). Broader visual orientation tuning in patients with schizophrenia. *Front. Hum. Neurosci.* 5:127 10.3389/fnhum.2011.00127 PMC3208208 22069385

[B45] Ruxton G. (2006). The unequal variance t-test is an underused alternative to Student’s t-test and the Mann–Whitney U test. *Behav. Ecol.* 17 688–690 10.1093/beheco/ark016

[B46] Schechter I. Butler P. Jalbrzikowski M. Pasternak R. Seperstein A. M. Javitt D. C. (2006). A new dimensión of sensory dysfunction: stereopsis deficits in schizophrenia. *Biol. Psychiatry* 60 1282–1284 10.1016/j.biopsych.2006.03.064 16945346 PMC2901805

[B47] Selemon L. D. Rajkowska G. Goldman-Rakic P. S. (1995). Abnormally high neuronal density in the schizophrenic cortex: a morphometric analysis of prefrontal area 9 and occipital area 17. *Arch. Gen. Psychiatry* 52 805–818 10.1001/archpsyc.1995.03950220015005 7575100

[B48] Self M. W. Lorteije J. A. M. Vangeneugden J. Van Beest E. H. Grigore M. E. Levelt C. N. (2014). Orientation-tuned surround suppression in mouse visual cortex. *J. Neurosci.* 34 9290–9304 10.1523/JNEUROSCI.5051-13.2014 25009262 PMC6608354

[B49] Serrano-Pedraza I. Grady J. P. Read J. C. A. (2012). Spatial frequency bandwidth of surround suppression tuning curves. *J. Vis.* 12 1–11 10.1167/12.6.24 PMC3927055 22715195

[B50] Serrano-Pedraza I. Romero-Ferreiro V. Read J. C. A. Diéguez-Risco T. Bagney A. Caballero-González M. (2014). Reduced visual orientation-surround suppression in schizophrenia shown by measuring contrast detection thresholds. *J. Vis.* 14:1406 10.1167/14.10.1406 PMC4261701 25540631

[B51] Sierra-Vazquez V. Serrano-Pedraza I. Luna D. (2006). The effect of spatial-frequency filtering on the visual processing of global structure. *Perception* 35 1583–1609 10.1068/p5364 17283927

[B52] Skottun B. Skoyles J. (2007). Contrast sensitivity and magnocellular functioning in schizophrenia. *Vision Res.* 47 2923–2933 10.1016/j.visres.2007.07.016 17825350

[B53] Slaghuis W. L. (1998). Contrast sensitivity for stationary and drifting spatial frequency gratings in positive- and negative-symptom schizophrenia. *J. Abnorm. Psychol.* 107 49–62 10.1037/0021-843X.107.1.49 9505038

[B54] Smith M. (2006). Surround suppression in the early visual system. *J. Neurosci.* 26 3624–3625 10.1523/JNEUROSCI.0236-06.2006 16597714 PMC6674136

[B55] Solomon S. G. White A. J. R. Martin P. R. (2002). Extraclassical receptive field properties of parvocellular, magnocellular, and koniocellular cells in the primate lateral geniculate nucleus. *J. Neurosci.* 22 338–349. 11756517 10.1523/JNEUROSCI.22-01-00338.2002 PMC6757604

[B56] Tadin D. Kim J. Doop M. L. Gibson C. Lappin J. S. Blake R. (2006). Weakened center-surround interactions in visual motion processing in schizophrenia. *J. Neurosci.* 26 11403–11412 10.1523/JNEUROSCI.2592-06.2006 17079669 PMC6674537

[B57] Tibber M. Anderson E. Bobin T. Antonova E. Seabright A. Wright B. (2013). Visual surround suppression in schizophrenia. *Front. Psychol.* 4:88 10.3389/fpsyg.2013.00088 PMC3584288 23450069

[B58] Tiippana K. Näsänen R. (1999). Spatial-frequency bandwidth of perceived contrast. *Vision Res.* 39 3399–3403 10.1016/S0042-6989(99)00057-7 10615504

[B59] Treutwein B. (1995). Adaptive psychophysical procedures. *Vision Res.* 35 2503–2522 10.1016/0042-6989(95)00016-X 8594817

[B60] Uhlhaas P. J. Mishara A. L. (2007). Perceptual anomalies in schizophrenia: Integrating phenomenology and cognitive neuroscience. *Schizophr. Bull.* 33 142–156 10.1093/schbul/sbl047 17118973 PMC2632288

[B61] Wassef A. Bajer J. Kochan L. D. (2003). GABA and schizophrenia: a review of basic science and clinical studies. *J. Clin. Psychopharmacol.* 23 601–640 10.1097/01.jcp.0000095349.32154.a5 14624191

[B62] Webb B. S. Dhruv N. T. Solomon S. G. Tailby C. Lennie P. (2005). Early and late mechanisms of surround suppression in striate cortex of macaque. *J. Neurosci.* 25 11666–11675 10.1523/JNEUROSCI.3414-05.2005 16354925 PMC6726034

[B63] Woods S. W. (2003). Chlorpromazine equivalent doses for the newer atypical antipsychotics. *J. Clin. Psychiatry* 64 663–667 10.4088/JCP.v64n0607 12823080

[B64] Yang E. Tadin D. Glasser D. M. Hong S. W. Blake R. Park S. (2013). Visual context processing in schizophrenia. *Clin. Psychol. Sci.* 1 1–11 10.1177/2167702612464618 PMC3756604 23997995

[B65] Yoon J. H. Maddock R. J. Rokem A. Silver M. A. Minzenberg M. J. Ragland J. D. (2010). GABA concentration is reduced in visual cortex in schizophrenia and correlates with orientation-specific surround suppression. *J. Neurosci.* 30 3777–3781 10.1523/JNEUROSCI.6158-09.2010 20220012 PMC2846788

[B66] Yoon J. H. Rokem A. S. Silver M. A. Minzeenberg M. J. Ursu S. Ragland J. D. (2009). Diminished orientation specific surround suppression of visual processing in schizophrenia. *Schizophr. Bull.* 35 1078–1084 10.1093/schbul/sbp064 19620601 PMC2762622

